# Postoperative amyloidosis of tongue base: Extremely rare complication after multilevel sleep surgery

**DOI:** 10.1002/ccr3.3812

**Published:** 2021-01-24

**Authors:** Ahmed Bahgat, Claudio Vicini

**Affiliations:** ^1^ Department of Otorhinolaryngology Alexandria University Alexandria Egypt; ^2^ Department of Head‐Neck Surgery, Otolaryngology, Head‐Neck and Oral Surgery Unit Morgagni Pierantoni Hospital Azienda USL della Romagna Forlì Italy

**Keywords:** amyloidosis, multilevel surgery, obstructive sleep apnea, postoperative complication, tongue base surgery

## Abstract

Amyloidosis is to be considered in the differential diagnosis of postoperative edema of tongue base after its ablation. It might be triggered by surgical trauma. After establishment of diagnosis, cause of secondary amyloidosis should be excluded.

## Graphical abstract

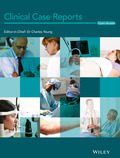

## 1 INTRODUCTION

Amyloidosis is a rare disorder where there is accumulation of pathologic deposits of amyloids in tissues. The amyloids are protein polymers formed of identical monomer units. Pathological amyloids are usually formed from misfolded proteins. The deposition of amyloids occurs either intracellularly or extracellularly alter the normal function of organs.^1^

Amyloidosis can be classified according to clinicopathological criteria as follows: (a) primary systemic amyloidosis, with completely normal laboratory and radiological investigations; (b) secondary systemic amyloidosis caused by chronic disease, such as tuberculosis or rheumatoid arthritis; (c) Hereditary systemic amyloidosis associated with multiple myeloma; and (d) localized amyloidosis with no evidence of systemic amyloidosis or underlying chronic disease.

Head and neck amyloidosis can be either localized or part of systemic affection; localized amyloidosis usually affects larynx and trachea.^2^ Tongue involvement is common in systemic amyloidosis either diffuse as macroglossia or localized. Localized tongue amyloidosis is extremely rare.^2^ This study presents a rare case of localized amyloidosis at the level of the tongue base following tongue base ablation surgery in the setting of multilevel surgery for management of severe obstructive sleep apnea patient.

## 2 CASE REPORT

A 48‐year‐old man with a history of loud snoring and severe obstructive sleep apnea (OSA) as diagnosed by Polysomnography (Apnea/hypopnea index AHI of 58/h (normal < 5/h)). He was operated by septoplasty, inferior turbinectomy, tonsillectomy, uvulopalatopharyngoplasty (UPPP), and coblation tongue base ablation at one setting one year before presentation. The patient had a tough postoperative course, severe pain, edema of the neck, and edema of tongue base. The patient suffered from dysphagia, difficult breathing, change of voice and persistent snoring, and OSA after surgery.

**Figure 1:**
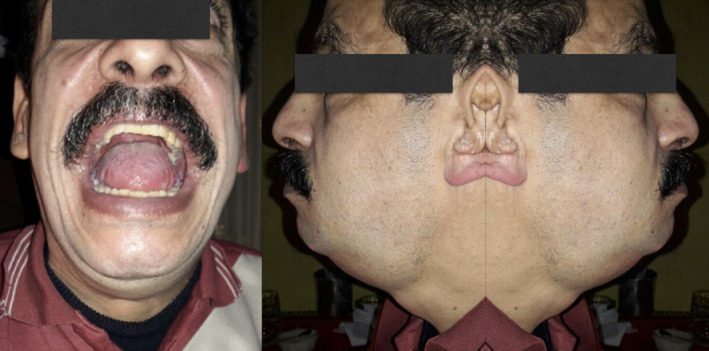
Clinical presentation of the patient showing firm neck edema and firm tongue edema with limited mouth opening

At presentation, the patient had diffuse submental and submandibular firm neck edema, limited mouth opening, and firm edema of tongue base with limited tongue movement (Figure 1). A flexible endoscopy was performed, revealing some retropalatal adhesions, diffuse edema of the tongue base, adhesions of lateral border of tongue base with lateral oropharyngeal walls limiting full tongue movements, but otherwise normal upper aerodigestive tract with no obstructive lesions.

**Figure 2:**
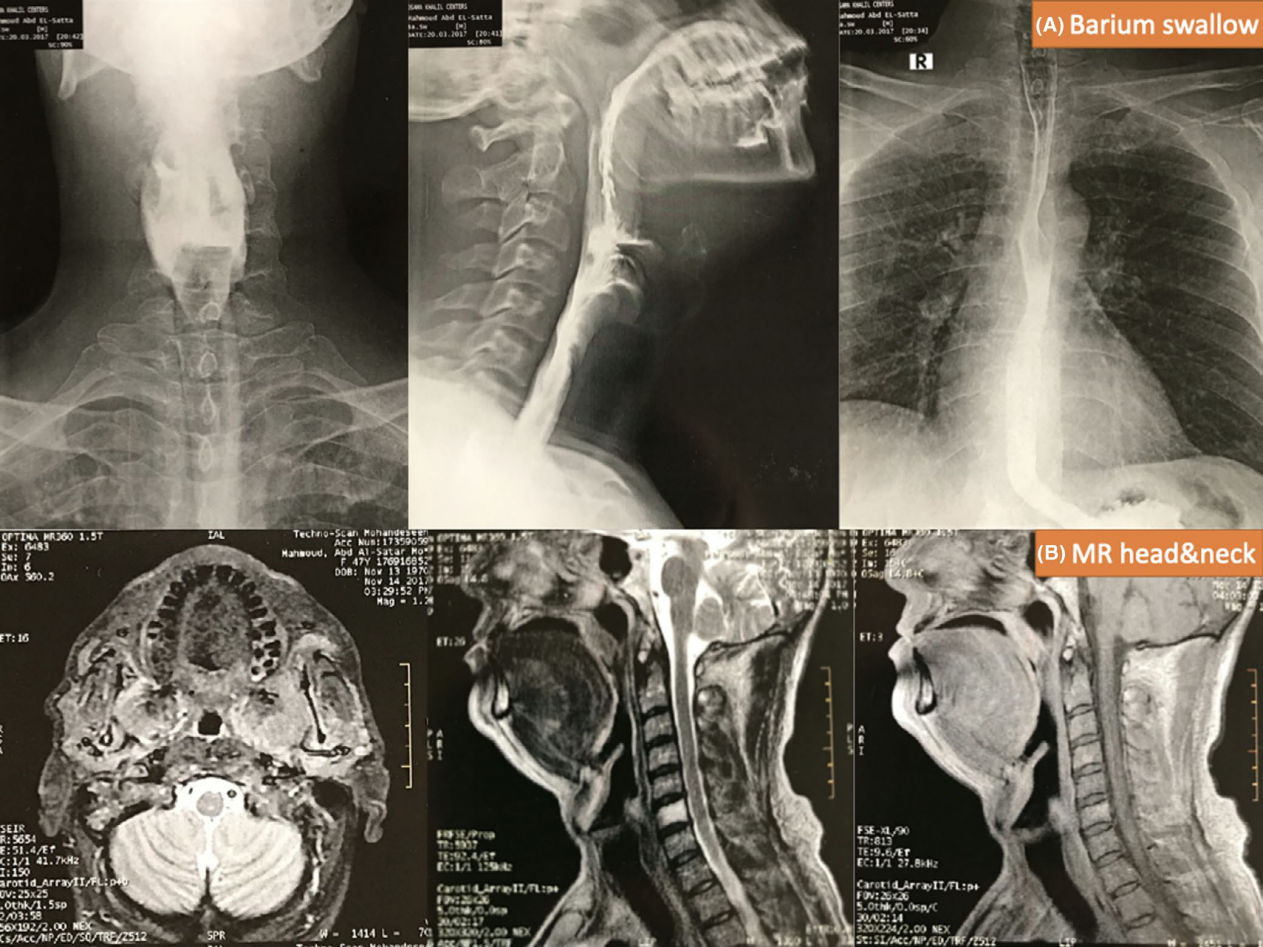
Radiological investigations of the patient; A, Barium swallow and B, MRI of the head and neck region

Radiological evaluation included barium swallow but showed normal barium decent through the pharynx and esophagus. CT scan of neck showed only submental and submandibular edema with mural thickening of the pharyngeal walls. Those findings were confirmed by head and neck MRI with contrast, it revealed retropalatal adhesions, bilateral symmetrical swollen pterygoid muscles “pterygoid rhabdomyolysis” as described by radiologists, causing oro‐ and hypo‐pharyngeal luminal narrowing (Figure 2).

Drug induced sedation endoscopy (DISE) was performed to confirm diagnosis; and taking punch biopsy from tongue base and fine‐needle aspiration cytology (FNAC) from submental and submandibular edema tissue while patient is sedated. Biopsy revealed fibrous tissue with amorphous eosinophilic materials, no malignancy, and no lymphoproliferative process. Further staining of the tissue with Congo red stain; and diagnosis was confirmed to be “Amyloidosis of tongue base”.

Patient was referred to hematologist to identify the etiology of amyloidosis. Patient was investigated by complete lab work with immunological tests and bone marrow biopsy. Bone marrow biopsy revealed interstitial increase in plasma cells and lymphocytes; immunohistochemical staining (IHC) showed few scattered CD20 positivity with 50%‐60% CD138 positive; and a picture was suggestive of plasma cell myeloma as a causative underlying etiology of amyloidosis. Patient started chemotherapy and steroids, and he showed improvement in swallowing and less edema of the tongue; however, he was put on CPAP therapy for residual OSA.

## 3 DISCUSSION

Amyloidosis can be classified into systemic and localized, and can be classified according to the type of deposited fibrinogen into immunoglobulin light‐chain amyloidosis (AL), amyloid A amyloidosis (AA), β2 microglobulin amyloidosis (Aβ2M), and transthyretin amyloidosis (ATTR).^3^ In systemic amyloidosis, the amyloid is deposited away from the site where it is produced, and it is transported via the circulatory system to the site of deposition. In localized amyloidosis, the location of amyloid production and deposition are the same. It is mainly caused by the AL amyloid, which is produced and deposited in local sites.^4^ The exact etiology of localized amyloidosis is not yet known.

When amyloidosis is suspected, it requires tissue biopsy under local anesthesia and microscopic examination [using hematoxylin and eosin (H&E), and Congo red stains] are usually sufficient to establish a diagnosis. The next step is to exclude other organ involvements in systemic amyloidosis, then exclude underlying systemic disease (eg, chronic inflammatory arthritis, tuberculosis, familial Mediterranean fever, and Crohn's disease). Finally, it is important to establish the subtype of amyloidosis. This is usually tested using serum or urine immunofixation electrophoresis to search for a clonal disorder.^5^

## 4 CONCLUSION

Recommendations regarding amyloidosis diagnosis are as follows: (a) Congo red stain is currently the gold standard for amyloid detection and (b) the type of amyloid must be identified microscopically or immunohistochemically, not solely on clinical or DNA studies. Cooperation with other medical specialties is crucial for correct and early diagnosis.

## CONFLICT OF INTEREST

None declared.

## AUTHOR CONTRIBUTIONS

AB: performed sleep endoscopy, tongue base biopsy, and FNAC from submental tissue edema. CV: examined the patient and recommended doing biopsy, and he was a major contributor in writing the manuscript. All authors read and approved the final manuscript.

## ETHICAL APPROVAL

This study was approved by the ethics committee of Alexandria University. The participation in the study was approved by the patient.

## Data Availability

The data that support the findings of this study are available on request from the corresponding author. The data are not publicly available due to privacy or ethical restrictions.

## References

[1] Buxbaum JN . The systemic amyloidoses. Curr Opin Rheumatol. 2004;16(1):67‐75. 14673392 10.1097/00002281-200401000-00013

[2] Musat G , Evsei A , Calina D , et al. Rare amyloidoma of the tongue base: a case report and review of the literature. Mol Clin Oncol. 2020;12(3):258‐262. 32064103 10.3892/mco.2020.1972 PMC7016517

[3] Mollee P , Renaut P , Gottlieb D , Goodman H . How to diagnose amyloidosis. Intern Med J. 2014;44(1):7‐17. 24024789 10.1111/imj.12288

[4] Pasternak S , White VA , Gascoyne RD , Perry SR , Johnson RL , Rootman J . Monoclonal origin of localised orbital amyloidosis detected by molecular analysis. Br J Ophthalmol. 1996;80(11):1013‐1017. 8976732 10.1136/bjo.80.11.1013 PMC505682

[5] Deng J , Chen Q , Ji P , Zeng X , Jin X . Oral amyloidosis: a strategy to differentiate systemic amyloidosis involving the oral cavity and localized amyloidosis. Oral Dis. 2019;25(3):670‐675. 29667278 10.1111/odi.12870

